# Our Bureau of Information

**Published:** 1911-12-23

**Authors:** 


### OUR BUREAU OF INFORMATION.

### Brotherhood Work.

### Ladies' Auxiliaries.

There is work for all in the world of charity, but all are not fitted for the same work. This is a statement so obvious as to be a platitude, but we venture on it because we hope to show one or two ways in which those can help who have hitherto refrained from charitable work, not from lack of good will so much as from lack of knowledge. Under these we may give as examples the work of Ladies' Auxiliaries to hospitals. The large tasks of hospital administration are generally left to men, and, as a rule, the large subscriptions come from men. But there is a vast number of small subscriptions which can be obtained if only people will take the trouble. These small sums—the half-crowns, shillings, and sixpences—involve far more labour in collection than the pounds and guineas, and institutions cannot afford to maintain a salaried collector for them, while it would not be remunerative for any collector to gather them in on commission. Unless voluntary help is available, these small sums, which in the mass total a large sum, will be lost to charity. While such work as this is peculiarly suitable for ladies with a sufficiency of leisure, it does not follow that those whose time is filled up by self-supporting work cannot help in it. Indeed, through such as they, sources may be tapped which would otherwise be left untouched. For example, it would be perfectly simple for a school teacher or a post-office clerk to collect from her colleagues trifling contributions, which might in the end come to something handsome. The League of Mercy has shown in London and the home counties what can be done if many workers unite in collecting shillings, and in many provincial towns the Ladies' Auxiliaries of the hospitals do similar work. But there is more to be done. Why should there not be a Ladies' Auxiliary to gather money for the district nurse and the cottage hospital in every village in the kingdom?

Of course this work, like every other, requires a head. There are many isolated units who lack the courage to institute such a work. They are afraid of the responsibility, they lack experience, they are shy of approaching people who may be older or richer than themselves. In such a case it is a good thing—though by no means an essential one—that any lady who is naturally in a leading position should organise a band of helpers. In it all questions of social precedence must be set aside. The recent political agitation among women has conclusively shown that where one aspiration is earnestly held in common the ordinary distinctions of social life are of very small account. And surely a unity which can be achieved for a political aim should be easy to attain in the cause of charity. Let us add, however, that there is one person—usually a very competent one—who ought not to be asked to organise an auxiliary. We mean the matron of the hospital, whose duties are always sufficiently absorbing and multifarious without having thrust upon her the management of a scheme which someone else can work quite as well. But if the governing body includes any ladies, one of them would be a suitable head, or, failing such, the wife of one of the Governors. Someone who has a personal, or all but personal, knowledge of hospital work and hospital needs may be more competent to organise an auxiliary than the most highly-placed or richest woman in the neighbourhood.

#### When the Patient Goes Out.

There is another work which ladies can do admirably. All nurses can tell how grieved they often are when, having brought a patient back to health, they see him go out clad in the thin and insufficient clothing in which he entered the hospital, and the insufficiency of which may have been one cause of his illness. It seems so useless to bring a person back to health if one has not the means of keeping him well. Every matron longs for a supply of clothing to give to necessitous patients, and this is a thing to which a Ladies' Auxiliary—or Samaritan Fund, as it is sometimes called—can contribute effectively. From our own distribution of garments we know how gratefully matrons and nurses welcome such gifts. And surely there are very few women who cannot give a garment a year to the poor. Nay, it need not even be a new garment. Old and worn things can be utilised wonderfully if someone—who, perhaps, can give nothing else—will give a little time to mending and patching them, and sometimes turning two thin garments into one solid and substantial one. We may mention that there is always a place for outgrown baby clothing in our maternity hospitals, which, unfortunately, do not get much aid from general charity. Shrunken flannels, shabby gowns and shawl may be a great boon to some helpless little creature whose lines, at the best, have not fallen in pleasant places.

#### The Etceteras of Dress.

But one precaution is desirable in regard to gifts of old clothes—that they should be received anonymously. Sensitive people, who are themselves compelled to wear their clothes to the last verge of decency, do not like to think of some richer person criticising the shabbiness of the things which are all that they can spare. If no one, not even the lady who receives the garments, need know from whom they come, many would be willing to give who are now restrained by a quite comprehensible sensitiveness. And with intelligence and good will something can be contrived from even the shabbiest things, for the best part of a large garment can be used to patch another, or to make something for a child. It may be remembered, too, that, while under-garments are always needed, there is also a use for the little *etceteras of dress.* Old hats, ribbons, and flowers may be utilised to make something that will give a woman that better appearance which will help her to get work. Old gloves, collars, and belts are not to be despised. And one last word—men's clothing, both under and outer garments, are always exceptionally welcome. They cost, even at second-hand dealers, more than the poor can spare, and a gift of them may be the greatest of blessings to a honest man who has been stricken down in the battle of life.

### Needs and Helpers.

#### An Effective Sputum Destructor.

"Forward" writes: I am most anxious to know what kind of sputum destructor has been found the most efficient for dealing with the sputum of consumptive patients, at what sanatorium it is in use, what firms supply it, and what is the cost?

"R. M. O." writes in answer: There is a destructor and cleaner combined—the "Meathop"—which acts admirably, but is quite unjustifiable from its expense. What "Forward" should do is to consult his engineers, and get made a tin tank, of suitable size, fitted with receptacles. Into this lead a steam-pipe, and when the mugs come down from the wards, empty the bulk of the expectoration into a can placed on paper, depositing both can and paper in the boiler furnace. Then fill the tin tank with lukewarm water, put the mugs in, turn the steam on, and leave for an hour. After this, a wipe with a rag and a little "Monkey Brand" soap is all that is necessary. If combustible cardboard mugs be obtained from the "Papperchief" firm—Messrs. G. Fingerhut, Alte Jakobstrasse, Berlin—it leaves only pocket flasks to be dealt with. For a fifty-bed sanatorium such mugs would cost about £18 a year, and the saving in labour—repulsive labour—is worth it to most places.

### Scarlet Fever Hospitals.

"Deutscher" writes: Can you give me some information about any other hospitals for scarlet fever cases than those large ones under the rates—I mean the Metropolitan Asylums Board's hospitals. They are very good, but very large, and, of course, all sorts of people go there, as they are free. But is there not a place where better-class children could go and pay a little?

(To meet the needs of just that class of case there is the excellent institution in the Liverpool Road, Islington, known as the London Fever Hospital. This hospital takes in all kinds of infectious diseases, but principally scarlet fever, diphtheria, and measles. The accommodation is of two kinds: (1) In the wards at a fee of three guineas for the whole time of the patient's stay, and (2) in private rooms at four guineas a week. Further particulars may be obtained from the Secretary. The hospital authorities fetch the patient in a brougham-ambulance, and a nurse is sent with it. The institution is well equipped, and the wards are quite up-to-date. There are visiting physicians and two resident medical officers.)

### "Totally Incapacitated and Paralysed."

"C. J." writes: Can I obtain assistance for my husband, who is now totally incapacitated and paralysed? He has been operated on for cerebral tumour.

(The Royal Hospital for Incurables at Putney gives pensions of £20 a year to incurable cases where the relatives are willing to have the patient at home. This would seem to be the kind of relief you would desire for your husband. As, however, these pensions are obtainable only through the votes of subscribers, the process of getting them is often a long and weary one. Still, as you are, as you tell us, a C.M.B. nurse, you will come into contact with many people, some of whom may have votes, and persons of influence connected with your husband's former work may be able to help you. It must be remembered, also, that in these institutions, where admission or other benefits are obtained by votes, there is a great deal done in the exchanging of votes for one institution for votes for another. Thus you might meet some sympathiser who has not votes for this institution, but has them for another, for which there is no candidate in whom he is interested. Such persons might give you the right to exchange these votes for votes for the Hospital for Incurables. A great deal of this is done, even on election days. The first thing to do is to get a list of subscribers, and see if you know or can reach any of these. After that, you must be persistent and energetic. Mention your desire to everyone with whom you come in contact. You never know who will be able to help you.)

### Letter-box Answers.

*Mrs. B.*: We do not prescribe.

*W. S., Leytonstone*: We have read your long and rather rambling statement of your case. You seem to have consulted four doctors, at three different hospitals, regarding a weakness of the ankle left after a broken leg had been set. Three of them gave one diagnosis, and the fourth another. You preferred the opinion of the last, and underwent an operation, the results of which have not satisfied you. It is obvious that in deciding one opinion out of four the onus of choice lies on you; nor does it appear—at least, so far as we can judge from your statement—that any of the surgeons promised that your ankle would be as strong as it was originally. Your disappointment is natural, but we cannot discover any grounds for blaming those whom you consulted.

